# Variation in the nuclear effects of infection by different human rhinovirus serotypes

**DOI:** 10.3389/fmicb.2015.00875

**Published:** 2015-08-24

**Authors:** Erin J. Walker, Lora M. Jensen, Sarah Croft, Reena Ghildyal

**Affiliations:** Centre for Research in Therapeutic Solutions, Faculty of Education, Science, Technology and Mathematics, University of Canberra, CanberraACT, Australia

**Keywords:** human rhinovirus, nuclear transport, nucleoporin, serotype, viral proteases

## Abstract

Human rhinovirus (HRV) is a positive sense RNA virus, which, despite replicating in the cytoplasm, has a significant impact on nuclear transport and nuclear localization of host proteins. A number of studies have identified differences between HRV serotypes, with respect to host response, protease activity and replicative ability. Here we report the sero-specific effects of two group-A HRV serotypes, the minor group HRV2 and the major group HRV16, on nuclear transport and nuclear protein localization. Using Western analysis, immunofluorescence and real time PCR, we show that HRV2 replicates at a faster rate than HRV16, which correlates with earlier production of viral proteases and disruption of host nuclear transport. There is significant variation in the nuclear effects of different rhinovirus species, which in turn may impact disease progression and patient response.

## Introduction

Human rhinovirus (HRV) is a positive sense RNA virus within the *Enterovirus* genus, which belongs to the *Picornaviridae* family. There are >100 strains of HRV, which have variously been categorized by their response to anti-viral compounds (Andries et al., 1990), the specific cell receptor used for entry into host cells [major group: intercellular adhesion molecule 1 (ICAM1) or minor group: low-density lipoprotein receptor (LDLR)] (Vlasak et al., 2005), or more recently, by genomic sequence analysis (currently categorized into HRV-A, HRV-B, and HRV-C) (Palmenberg et al., 2009).

Despite being a positive sense RNA virus with a wholly cytoplasmic replication cycle, HRV proteins are known to interact significantly with the host nucleus, both to alter and utilize host proteins for viral polyprotein production, as well as subverting the host immune response and shutting down host transcription and translation. These processes are achieved via the activity of the virally encoded proteases, 2A^pro^ and 3C^pro^, and involve the cleavage of specific host nuclear and nuclear-pore proteins (Bushell et al., 2001; Gustin and Sarnow, 2002; Amineva et al., 2004; Watters and Palmenberg, 2011; Walker et al., 2013).

A number of studies have examined the differences between HRV serotypes, considering both protease activity as well as host cytokine response. A study of recombinant 2A^pro^ activity *in vitro* from different serotypes demonstrated that the protease activity of 2A against specific host cell proteins varies with HRV-A > HRV-C >> HRV-B (Watters and Palmenberg, 2011). In addition, *in vitro* cleavage of host nuclear proteins during infection with HRV16 (Group A) occurred earlier than cleavage by HRV14 (Group B) (Watters and Palmenberg, 2011). Others have examined the effect of HRV species on cytokine response, identifying significant variation in cytokine production associated with HRV serotype (Nakagome et al., 2014; Rajan et al., 2014). Furthermore, there is some clinical data to support the notion that HRV-A viruses cause more severe clinical disease compared to HRV-B viruses (Iwane et al., 2011; Lee et al., 2012).

The few published studies comparing major and minor HRV serotypes within the same group have identified reduced replicative ability in minor HRV serotypes compared to major HRV serotypes, as well as variation in disease severity and cytokine response (Wark et al., 2009; Schuler et al., 2014). Since it is evident that significant variation exists between HRV serotypes even within the same group, both in terms of viral protease activity and host response, we examined the effect of a major and minor group HRV-A virus on host nuclear transport. We found infection with the minor group HRV2 resulted in fully processed viral proteases evident earlier during infection compared to HRV16, leading to earlier cleavage of host nuclear pore proteins. Interestingly, we found that infection with HRV2 results in cleavage of nucleolin as well as the relocalization of SC35, as has been previously described for HRV16. Finally we found the HRV2-induced relocalization of hnRNP-C1/C2 occurs at least 3 h prior to that induced by HRV16 infection, timing that correlates with more complete disruption of nuclear pore components.

## Materials and Methods

### Antibodies

The primary antibodies for the following proteins were used for Western analysis and immunofluorescence (IF): anti-Nup62 (BD Biosciences #610497, used at 1:2000 for Western), anti-Nup98 (Abcam #45584, used at 1:1000 for Western), anti-Nup153 (Abcam #96462, used at 1:1000 for Western), anti-eIF4G (BD Biosciences #610536, used at 1:1000 for Western), anti-PABP (Cell Signaling Technology #4992, used at 1:1000 for Western), anti-nucleolin (Abcam #22758, used at 1:3000 for Western), anti-hnRNP-C1/C2 (Santa Cruz #32308, used at 1:500 for IF), anti-SC35 (Sigma #4045, used at 1:500 for IF), anti-Sam68 (Santa Cruz #sc333, used at 1:500 for IF), and anti-α/β-tubulin (Cell Signaling Technology #2148, used at 1:1000 for Western). Antibodies to 3C^pro^ were kindly provided by S. Amineva (Madison, WI, USA; Amineva et al., 2004) and antibodies to dsRNA were kindly provided by S. Bowden (VIDRL, Melbourne, VIC, Australia).

### Cell Culture and Infection

Ohio-HeLa cells (provided by Bo Lin, Biota Holdings) and A549 cells (ATCC) were grown in high glucose DMEM supplemented with 10% heat inactivated Foetal Bovine Serum (FBS) and antibiotics (penicillin, streptomycin, neomycin) at 37°C in a humidified atmosphere of 5% CO_2_. For HRV infection of A549 cells, 20 mM MgCl_2_ was added to the infection media. Rhinovirus serotype 16 (HRV16) was a gift from E. Dick and W. Busse (Madison, WI, USA) and Rhinovirus serotype 2 (HRV2) was provided by Biota Holdings. Viral stocks were prepared by infecting subconfluent monolayers of Ohio-HeLa cells at a multiplicity of infection (MOI) of 1 by absorption for 1 h with occasional rocking, followed by replacement of the medium with fresh DMEM supplemented with 2% FBS and antibiotics. Once extensive cytopathic effects were observed, infected cultures were frozen at –80°C to release virus (Ghildyal et al., 2005). Cultures were thawed, vortexed and clarified of cellular debris by centrifugation for 15 min at 3,500 rpm. Infectious virus was titrated on Ohio-HeLa cells by standard TCID50 protocol and titre calculated using the Spearman–Karber equation (Mahy and Kangro, 1996).

### Western Analysis

Overnight subconfluent cultures of Ohio-HeLa or A549 cells with or without infection with HRV2 or HRV16 at an MOI of 1 (Ohio-HeLa cells) or 5 (A549 cells) were lysed at different times by incubation in RIPA buffer containing protease and phosphatase inhibitors (Roche) for 30 min on ice (Walker et al., 2013), prior to heating at 100°C for 5 min in Laemmli buffer (Hames, 1998). Cell lysates were subjected to SDS-polyacrylamide electrophoresis using pre-cast gradient (4–20%) or 10% acrylamide gels (Bio-Rad, TGX gels) followed by Western transfer to nitrocellulose membranes in Tris-Glycine-ethanol buffer (25 mM Tris-HCl, 192 mM Glycine, 20% Ethanol) for 90 min at 400 mA. Blots were stained with Ponceau S (Sigma) to confirm transfer and blocked for 1 h in 4% skim milk (Diploma) in PBS (10 mM Na_2_HPO_4_, 1.7 mM KH_2_PO_4_, pH 7.2, 2.7 mM KCl, 137 mM NaCl), prior to incubation with different primary antibodies diluted in 1% skim milk in PBS-T (PBS containing 0.1% Tween 20) overnight at 4°C with rocking. After washing in PBS-T, blots were incubated with species specific secondary antibodies conjugated to horseradish peroxidase diluted 1:5000 in 1% skim milk in PBS-T, followed by washing and detection of bound antibodies with Enhanced Chemiluminescence (ECL, Perkin Elmer). Protein bands were detected using the Licor OdysseyFc, and captured digital images were analyzed using ImageStudio to quantitate relative protein levels. Where required, blots were stripped to remove bound antibodies (2% SDS, 62.5 mM Tris-HCl pH 6.8, 114.4 mM β-mercaptoethanol) at 50°C for 10 min, washed in PBS-T, blocked in 4% skim milk in PBS and reprobed using different primary antibodies as required.

### Immunofluorescence

Overnight subconfluent monolayers of Ohio-HeLa or A549 cells grown on glass coverslips (Proscitech, #1) were infected with HRV16 or HRV2 at an MOI of 1 (Ohio-HeLa cells) or 5 (A549 cells) or left uninfected (mock) and fixed with 4% formaldehyde in PBS followed by permeabilization of cell membranes with 0.2% Triton X-100 in PBS at various times post infection (p.i.) (Ghildyal et al., 2005). Cells were incubated with primary antibodies diluted in PBS for 30 min, washed twice in PBS, incubated with species specific secondary antibodies conjugated to CF 488 (Biotium) or Alexa Fluor 568 diluted 1:1000 in PBS for 30 min, and then washed twice in PBS and mounted using ProLong Gold mounting media with DAPI (Invitrogen). Digitized fluorescent cell images were collected using a Nikon Ti Eclipse confocal laser-scanning microscope (CLSM) with Nikon 60×/1.40 oil immersion lens (Plan Apo VC OFN25 DIC N2; optical section of 0.5 μm) and the NIS Elements AR software. Quantitative analysis of the fluorescence signal in the nucleus (Fn) and cytoplasm (Fc) was performed using ImageJ.

### RNA Isolation and Real Time PCR Analysis

Overnight subconfluent cultures of Ohio-HeLa or A549 cells were infected as described above and RNA was collected using Tri-Reagent (Sigma). Two samples were collected for each infection time point. At the indicated times, media was completely removed and 500 μL of Tri-Reagent was added to each 35 mm dish. Cell lysate was collected in 1.5 mL Eppendorf tubes and frozen at –80°C for subsequent analysis. Samples were thawed on ice, 100 μL of chloroform was added and samples were vortexed briefly, then incubated on ice for 2 min. Samples were then centrifuged at 10,000 rpm for 15 min at 4°C. The aqueous layer was transferred to a new tube and an equal volume of isopropanol was added. Samples were vortexed and the RNA allowed to precipitate overnight at –20°C. The next day, RNA samples were centrifuged at 10,000 rpm for 10 min at 4°C to pellet the RNA. The pellets were washed with 1 mL of 75% ethanol in DEPC water, vortexed and centrifuged at 7,500 rpm for 5 min at 4°C. All ethanol was removed and pellets were left to dry at room temperature. Dried pellets were resuspended in 15–30 μL of DEPC-water, depending on the size of the pellet. RNA concentration was determined using a NanoDrop 1000 instrument. To generate cDNA, 1 ug from each sample/time point was combined to transcribe a total of 2 ug of RNA per time point. Reverse transcription reactions were performed using the High Capacity cDNA Reverse Transcription kit (Applied Biosystems), following the manufacturer’s instructions. Real time PCR was performed on a Bio-Rad CFX96 instrument, using SsoAdvanced Universal SYBR Green Supermix (Bio-Rad) as per the manufacturer’s instructions, with the following additions: cDNA samples were diluted 1:50 prior to analysis, and the standard curve was generated by combining cDNA from each sample, then diluted fivefold to generate a 5 sample standard curve. Samples were amplified in duplicate and resultant real time PCR data was analyzed using CFX Manager (Bio-Rad). PCR primer sequences for viral RNA and GAPDH are available on request.

### Statistical Analysis

GraphPad Prism 6 was used for analyses; a two-tailed Mann–Whitney test was used to assess significant differences in Fn/c values for hnRNP-C1/C2 and Sam68 localization, compared to mock samples.

## Results

### Production of HRV2 Viral Proteins Occurs Earlier during Infection Compared to HRV16

**FIGURE 1:**
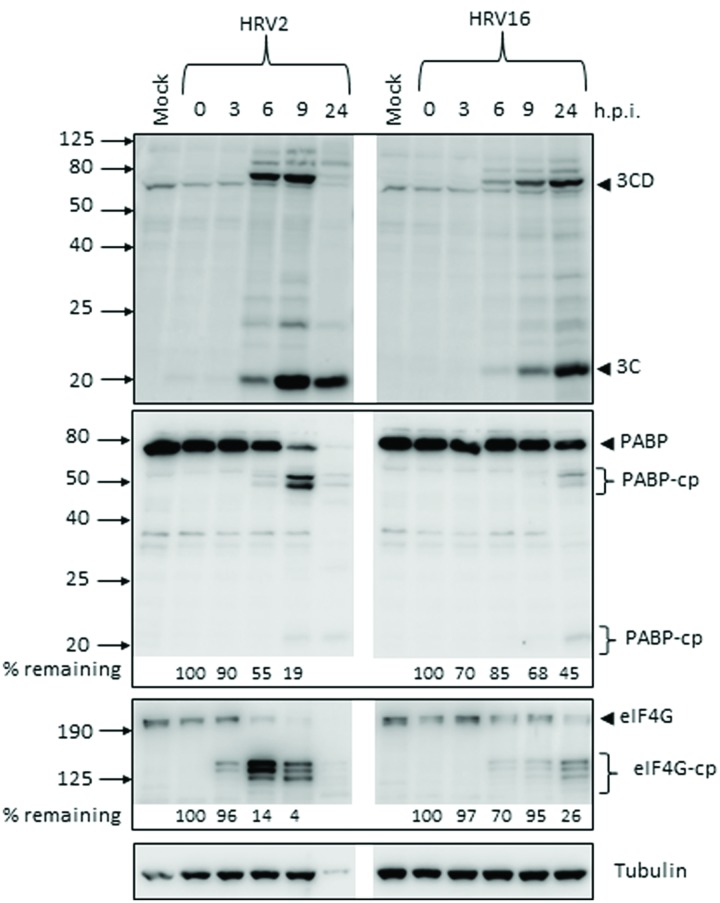
**Active proteases are produced at different times after infection with human rhinovirus 2 or 16 (HRV2 or HRV16).** Ohio-HeLa cells were infected without (mock) or with HRV2 or HRV16 (MOI of 1) and cells lysed using RIPA buffer containing protease and phosphatase inhibitors at the time points shown. Cell lysates were subjected to SDS-PAGE on 4–20% gradient gels (for anti-3C detection) or 10% gels (remaining antibodies) and Western analysis using the indicated primary antibodies/horseradish peroxidise-conjugated secondary antibodies and enhanced chemiluminescence (Perkin Elmer). The approximate protein size (kDa) is shown on the left and the specificity of the antibodies is indicated on the right. Where cleavage products are observed, bands corresponding to full length proteins are indicated with arrowheads and cleavage products are indicated with brackets. cp, cleavage products; h.p.i, hours post-infection. Results for densitometric analysis of protein bands are shown below the relevant blots, where data were normalized to the corresponding values for tubulin, and are shown as the percent protein remaining relative to the corresponding values for the 0 h sample.

The production of the viral proteases 2A and 3C during the course of Ohio-HeLa infection with HRV2 and HRV16 was assessed by Western blot (**Figure 1**), with the percentage of full length protein remaining relative to 0 h.p.i (hours post infection) shown under the appropriate lane. Quantitation for HRV2 infection at 24 h.p.i is not shown, as there was a significant decrease in tubulin which caused skewing of the results. However, the overall trend is apparent. The production and activity of 3C^pro^ was assessed by direct detection with an anti-3C antibody as well as by monitoring the appearance of cleavage products for a known target of 3C^pro^, poly(A)-binding protein (PABP). The production and activity of 2A^pro^ was assessed by monitoring the appearance of cleavage products of a known 2A^pro^ target, eukaryotic initiation factor 4G (eIF4G).

Despite infecting Ohio-HeLa cells at the same MOI, production of active viral proteases was noticeably different between HRV2 and HRV16. In HRV2 infected cells, 3C^pro^ was detectable by 6 h.p.i, with the greatest amount of HRV2 3C^pro^ present at 9 h.p.i; this coincides with the level of full length PABP, where 55% remains at 6 h.p.i and only 19% at 9 h.p.i. In HRV16 infected cells, 3C^pro^ was detectable from 6 h.p.i, though at lower levels compared to HRV2 infected cells at the same time p.i. The greatest amount of HRV16 3C^pro^ is observed at 24 h.p.i, which coincides with the greatest quantity of PABP cleavage products.

A similar difference in also observed for 2A^pro^, where the appearance of eIF4G cleavage products (as a proxy for detecting 2A^pro^ directly) was observed from 3 h.p.i in HRV2 infection, while for HRV16 infection these cleavage products were observed from 6 h.p.i. In addition, the rate of eIF4G cleavage appeared to be faster in HRV2 infection, with only 4% of full length eIF4G remaining at 9 h.p.i, while 26% of full length eIF4G still remained at 24 h.p.i after HRV16 infection. Similar results were observed for A549 cells at 24 h.p.i, where 3C and 3CD can be detected for both HRV2 and HRV16 infection. Cleavage of eIF4G was only observed for HRV2 infection, a result that mirrors the time difference seen in Ohio-HeLa infection (**Supplementary Figure S1A**).

### HRV Protease Activity Against Host Nuclear Pore Proteins is Delayed in HRV16 Infection

**FIGURE 2:**
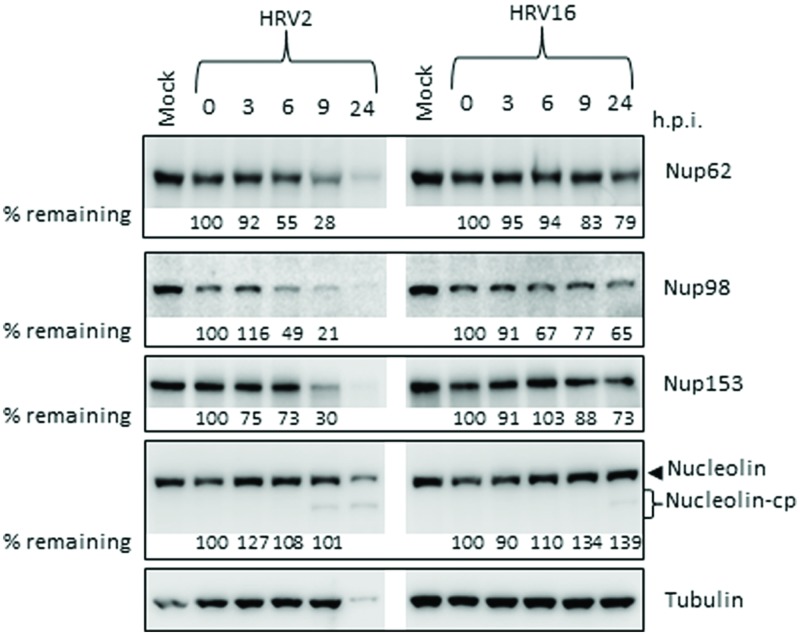
**Human rhinovirus protease activity occurs at different times after infection with HRV2 or HRV16.** Cell lysates were prepared and subjected to Western analysis as for **Figure 1**. The specificity of the antibodies is indicated on the right. Where cleavage products are observed, bands corresponding to full length proteins are indicated with arrowheads and cleavage products are indicated with brackets. cp, cleavage products; h.p.i, hours post-infection. Results for densitometric analysis of protein bands are shown below the relevant blots, where data were normalized to the corresponding values for tubulin, and are shown as the percent protein remaining relative to the corresponding values for the 0h sample.

Since there were obvious differences in the rate of protease production as well as in the proteolytic activity of the proteases from different serotypes, we next examined the effect of HRV2 and HRV16 infection on the cleavage of specific nucleoporins (Nups) in Ohio-HeLa cells, as well as on a nucleolar protein, nucleolin. Representative Western blots are shown in **Figure 2**, with the percentage of full length protein remaining relative to 0 h.p.i shown under the appropriate lane. HRV16-mediated cleavage of Nups 62, 98, 153, and nucleolin was delayed compared to HRV2. Analysis of Nup62 in HRV2 infected cells demonstrated that 55% of the protein remained at 6 h.p.i, and only 28% at 9 h.p.i. In contrast, HRV16-mediated degradation of Nup62 began between 9 and 24 h.p.i, as 79% of Nup62 was still apparent at 24 h.p.i.

Cleavage of Nup98 also began earlier in HRV2 infected cells, with results similar to those observed for Nup62, where approximately 50% of the full length protein had been cleaved by 6 h.p.i and only 21% remained at 9 h.p.i. In cells infected with HRV16, cleavage of Nup98 was apparent by 6 h.p.i, however, at 24 h.p.i, more than 50% of full length protein remained.

HRV2 appears to cleave Nup153 at a faster rate than HRV16, as approximately 70% of Nup153 was cleaved in HRV2 infected cells at 9 h.p.i, compared to less than 20% in HRV16 infected cells at the same time. Indeed, by 24 h.p.i, 27% of Nup153 (73% remaining) was cleaved in HRV16 infected cells. Together, these results demonstrate significant differences in the proteolytic activities of different HRV serotypes against specific host targets.

Finally, analysis of nucleolin showed that although there was no significant loss of full length protein after infection with either HRV2 or HRV16, a cleavage product was detected from 9 h.p.i in HRV2 and at 24 h.p.i in HRV16 infected cells. A similar result was observed for A549 cells at 24 h p.i., where a cleavage product can be observed in HRV2 infected cells, but not in HRV16 infected cells, again demonstrating the difference in proteolytic activity between the two viruses (**Supplementary Figure S1B**). The apparent increase in nucleolin during the course of Ohio-HeLa infection can be explained by our previous results (Walker et al., 2013), which showed that nucleolin is mislocalized from nucleoli into the nucleus during infection with HRV16 and therefore may be more easily detected.

### HRV Infection and Mislocalization of Nuclear Proteins

**FIGURE 3:**
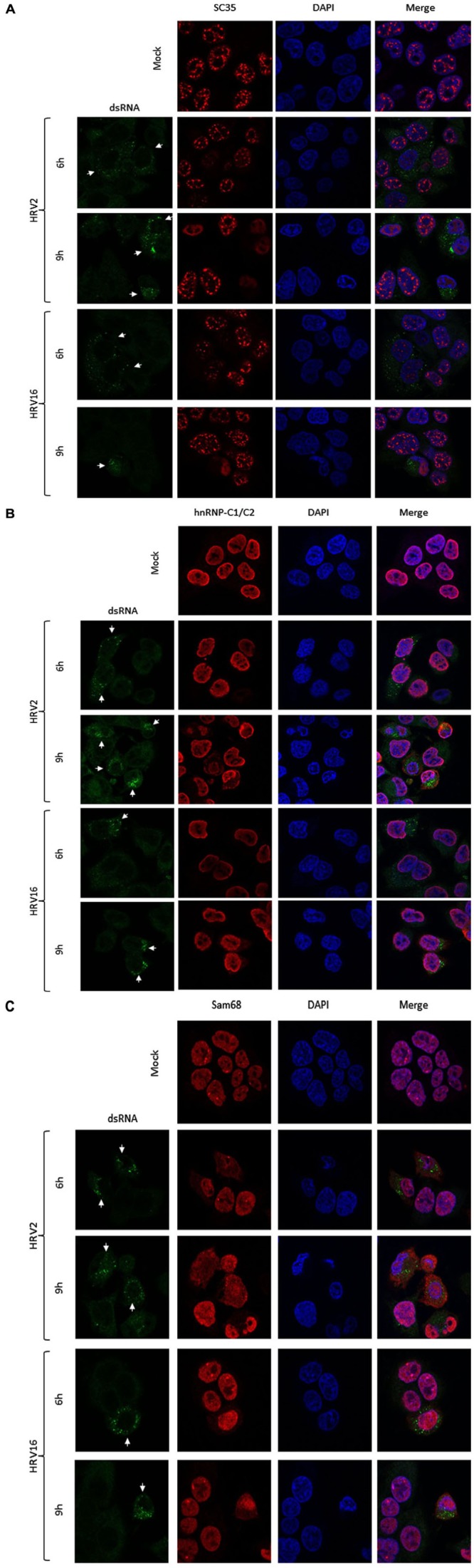
**HRV2 and HRV16 infection lead to mislocalization of nuclear proteins.** Ohio-HeLa cells grown on coverslips were infected without (mock) or with HRV2 or HRV16 as for **Figure 1**; cells were fixed at the indicated times and permeabilized, and then probed with **(A)** anti-dsRNA and anti-SC35 antibodies, **(B)** anti-dsRNA and anti-hnRNP-C1/C2 antibodies, or **(C)** anti-dsRNA and anti-Sam68 antibodies, followed by CF488 and Alexa-568 conjugated secondary antibodies. Coverslips were mounted in ProlongGold mounting media with DAPI. Fluorescence was imaged by CLSM (see Materials and Methods). White arrows indicate infected cells.

We next examined the effect of HRV2 and HRV16 infection on the nuclear localization of specific proteins, using IF to identify changes in localization. Ohio-HeLa cells were grown on coverslips and infected with HRV2 or HRV16 or mock infected, then fixed at indicated times post-infection. Cells were co-stained for dsRNA to identify infected cells and specific nuclear proteins as indicated (**Figure 3**). **Figure 3A** shows the effect of HRV infection on nuclear speckle protein SC35. At 6 h after HRV2 infection, the SC35 staining was fainter and less obvious, though the protein was still present in small nuclear speckles. By 9 h.p.i, SC35 had become diffuse throughout the nucleus. A similar effect was observed in HRV16 infected cells, where at 6 h.p.i SC35 speckles were fainter than mock infection, and by 9 h.p.i SC35 was diffuse throughout the nucleus. Thus, in contrast to results observed for HRV14 (Gustin and Sarnow, 2002), cleavage or degradation of the nuclear speckle protein SC35 was observed in both HRV2 and HRV16 infected cells.

**FIGURE 4:**
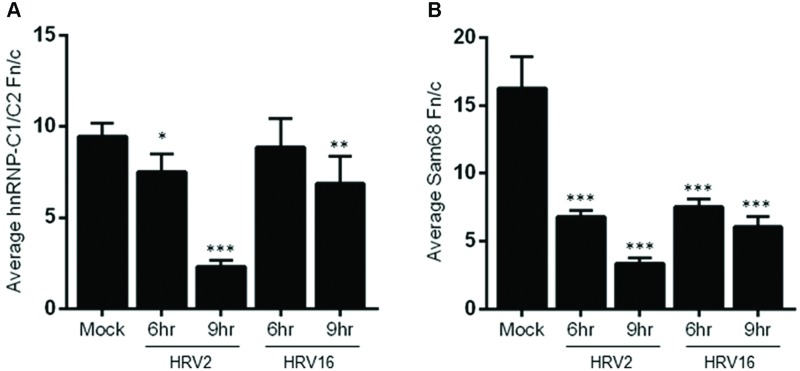
**Infection with HRV2 or HRV16 leads to significant mislocalization of nuclear proteins.** The nuclear and cytoplasmic fluorescence of infected and mock infected cells prepared as in **Figure 3** was quantitated using ImageJ, and the mean Fn/c values were plotted for **(A)** hnRNP-C1/C2 and **(B)** Sam68. Values are shown +SEM. Significance compared to mock values are shown as ^∗^*p* < 0.01, ^∗∗^*p* < 0.001, ^∗∗∗^*p* < 0.0001.

Examination of hnRNP-C1/C2 by IF (**Figure 3B**) showed limited nuclear mislocalization of hnRNP-C1/C2 at 6 h.p.i in HRV2 infected cells. However by 9 h.p.i, there was significant mislocalization of hnRNP-C1/C2 into the cytoplasm. Quantitation of nuclear compared to cytoplasmic fluorescence (Fn/c, **Figure 4A**) confirmed a moderately significant difference in localization at 6 h.p.i (*p* < 0.01) and a highly significant difference at 9 h.p.i (*p* < 0.0001) compared to mock infection. A similar trend was also observed for cells infected with HRV16, however, quantitation showed there was a significant decrease in Fn/c only at 9 h.p.i (*p* < 0.001) when compared to mock (**Figure 4A**).

**Figure 3C** depicts HRV2 and HRV16 infected cells stained for the nuclear protein Sam68. Mislocalization of Sam68 from the nucleus into the cytoplasm was apparent at all timepoints, and quantitation of nuclear compared to cytoplasmic fluorescence (Fn/c, **Figure 4B**) confirmed this observation, with a highly significant decrease in Fn/c (*p* < 0.0001) seen for both HRV2 and HRV16, at 6 and 9 h.p.i.

Together these results suggest that disruption of nuclear trafficking leading to mislocalization of nuclear proteins occurs early during infection with both HRV2 and HRV16, as SC35 and Sam68 were affected by 6 h.p.i in cells infected with either serotype. Disruption leading to mislocalization of hnRNP-C1/C2 appears to occur later during infection, as infection with HRV2 at 6 h.p.i led to a small change in nuclear localization compared to the more significant difference observed at 9 h.p.i for HRV2; infection with HRV16 had a more subtle effect on hnRNP-C1/C2 localization, with significant changes compared to mock only apparent at 9 h.p.i. While it could be argued that nuclear protein mislocalization is a result of apoptosis, we have treated Ohio-HeLa cells with Actinomycin D (5 μg/ml) to induce apoptosis, followed by staining for SC35, hnRNP-C1/C2 and Sam68 as described above; no changes in nuclear protein localization were observed during this treatment (data not shown).

We also examined whether HRV2 and 16 could replicate in A549 cells, and observed dsRNA in cells infected with both serotypes at 24 h p.i., indicating replication of the viral genome (**Supplementary Figure S1C**).

### Viral RNA Replication Occurs Earlier during Infection with HRV2

**FIGURE 5:**
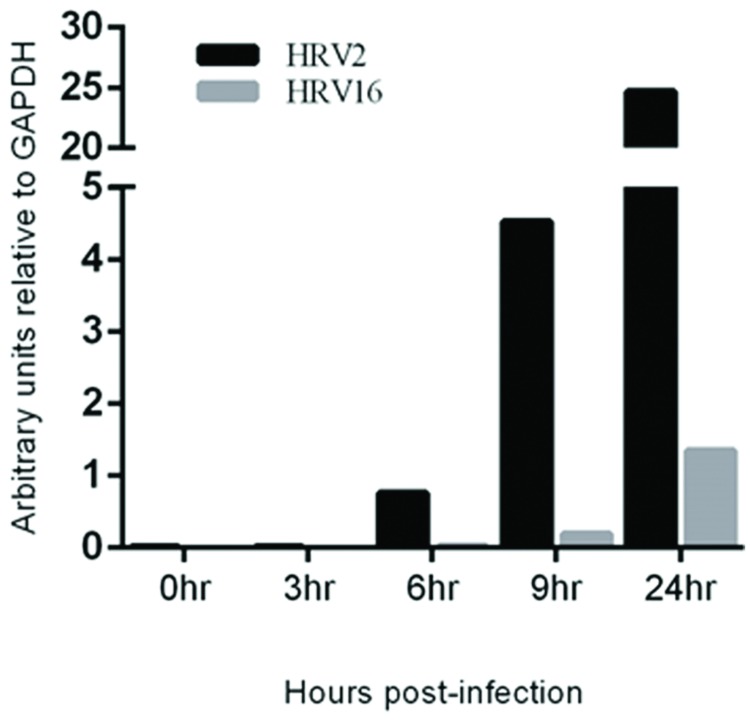
**Viral RNA replication is detectable earlier in HRV2 infection compared to HRV16 infection.** Real-time PCR was used to assess the quantity of viral RNA present in infected cells at the specified time points for HRV2 and HRV16 infection. Data is shown as arbitrary units relative to GAPDH.

To assess the rate of virus replication, the relative amount of viral RNA in cells infected with HRV2 or HRV16 at specified time points was measured. RNA was collected from HRV2 or HRV16 infected Ohio-HeLa cells, reversed transcribed and used in real-time PCR reactions, using primers specific for HRV viral RNA (Dagher et al., 2004) as well as GAPDH as a loading control. Data is shown as arbitrary units of viral RNA relative to GAPDH (**Figure 5**). These results show a steady increase in HRV2 viral RNA from ∼1 unit at 6 h.p.i to ∼4.5 units at 9 h.p.i and ∼25 units at 24 h.p.i. In contrast, viral replication after infection with HRV16 appeared to lag behind HRV2, with <1 unit detected at 9 h.p.i and ∼1.5 units detected at 24 h.p.i; these results correlate with the lag observed for protease activity against host proteins (**Figures 1** and **2**). Given the results shown in **Supplementary Figure S1**, we predict that it should also be possible to detect HRV genomes in infected A549 cells. Furthermore, since it appears that HRV2 is able to produce viral proteins and potentially assemble new virions more quickly than HRV16, we may also expect to observe less HRV2 viral RNA compared to that generated in HRV16 infected cells. Indeed, analysis of infected A549 cells at 24 h p.i. shows both HRV2 and HRV16 are able to replicate in these cells (**Supplementary Figure S2**) and there is more HRV16 viral RNA in cells compared to HRV2 viral RNA.

## Discussion

In the current study, we found the minor group HRV2 was able to replicate more efficiently than the major group HRV16 (**Figure 5**), which resulted in earlier production of viral proteases, concomitant with earlier cleavage of the target proteins PABP and eIF4G (**Figure 1**). In addition, the earlier production of viral proteases led to earlier and more complete cleavage of host nuclear pore proteins (Nups) as well as cleavage of the nucleolar protein, nucleolin (**Figure 2**). Despite the apparent lag in replication and therefore protease activity between HRV2 and HRV16, analysis of IF results (**Figures 3** and **4**) indicate that changes occur in the appearance of nuclear speckle protein SC35 by 6 h.p.i with HRV16, as well as significantly altering the nuclear localization of Sam68 at this time. In contrast, the mislocalization of nuclear protein hnRNP-C1/C2 occurred later, by 9 h.p.i for HRV2 infection and >9 h.p.i for HRV16 infection, possibly once complete disruption of the NPC has been achieved.

While picornavirus protease cleavage of PABP, eIF4G, and Nups is well established (Lamphear et al., 1993; Liebig et al., 1993; Gustin and Sarnow, 2002; Amineva et al., 2004; Kuyumcu-Martinez et al., 2004; Watters and Palmenberg, 2011), the effect on nucleolin beyond mislocalization (Gustin and Sarnow, 2002) has not been explored in detail. Here we show that infection with either HRV2 or HRV16 can lead to the presence of a cleavage product, similar to our previously reported results for HRV16 (Walker et al., 2013). The exact reason for nucleolin cleavage in HRV infection is unclear, as there are no reports of this event during infection with other viruses. Indeed, nucleolin has been reported as the cellular receptor for respiratory syncytial virus (Tayyari et al., 2011) and has been implicated as a required interacting protein in a number of other virus infections, including human cytomegalovirus (Strang et al., 2012), rabies virus (Oksayan et al., 2015), herpes simplex virus 1 (Greco et al., 2012), and a member of the picornavirus family, enterovirus 71 (Su et al., 2015). One possibility is that HRV infection initiates the apoptotic cascade in such a manner that cleavage of nucleolin is a downstream result, as nucleolin at the approximate size we observed has been reported to be cleaved by okadaic acid-induced apoptosis in cell lines (Kito et al., 2003), however, further investigation is required.

The mislocalization of Sam68 and alteration in the appearance of SC35 occurred as an early event during infection with both HRV2 and HRV16, before there was significant disruption of nuclear pore components in either HRV2 or HRV16 infection, suggesting that either a very small amount of viral protease activity is required to initiate these changes, or that early infection events prompt an additional pathway that contributes to the observed changes. That Sam68 is mislocalized during picornavirus infection has been well established (McBride et al., 1996; Gustin and Sarnow, 2002; Walker et al., 2013), however, HRV-initiated changes in SC35, an essential component of the spliceosome, have only been reported for HRV16 (Walker et al., 2013). In the current paper we also describe the same changes in HRV2 infection; this is in contrast to HRV14 or poliovirus infection (Meerovitch et al., 1993; Gustin and Sarnow, 2001; Gustin and Sarnow, 2002), where no changes in SC35 appearance were observed. While there is precedent for viruses that undergo alternative splicing, such as human papilloma virus (McFarlane and Graham, 2010) and human immunodeficiency virus (Maldarelli et al., 1998), to induce expression of SC35, the reason for HRV disruption of SC35 is unclear, beyond general disruption of the host cellular transcription machinery. It remains to be determined whether alterations in SC35 occur more generally in HRV infection, or if they are group specific and do not occur in Group B HRV.

The mislocalization of hnRNP-C1/C2 in picornavirus infection has been reported previously (Gustin and Sarnow, 2001; Gustin and Sarnow, 2002) and our results are consistent with these reports. Interestingly and in contrast to Sam68 and SC35, the timing of hnRNP-C1/C2 mislocalization varied between HRV2 and HRV16 by at least 3 hours. This delay in hnRNP-C1/C2 mislocalization better reflects the observed disruption of nuclear pore components, which are cleaved much earlier in HRV2 infected cells. Thus it is likely that complete disruption of the nuclear pore and nuclear trafficking is required before hnRNP-C1/C2 is displaced into the cytoplasm. The reason for this requirement is unclear, since hnRNP-C1/C2, at ∼40 kDa, is a similar size to both SC35 (35 kDa) and Sam68 (68 kDa) so all might be expected to diffuse into the cytoplasm at a similar time. Alternatively it is possible the strong nuclear retention signal carried by hnRNP-C1/C2 (Nakielny and Dreyfuss, 1996) enables these proteins to maintain their nuclear localization for longer during infection.

The impact of HRV serotype on pathogenesis has been examined by a small number of studies, which have found differences in the host immune response to major vs minor group HRV, as well as reduced cell viability after infection with minor group HRV (Wark et al., 2009; Schuler et al., 2014); we also observe greater cell death in cells infected with minor group HRV2 compared to major group HRV16 (**Supplementary Figure S3**). A study of asthma exacerbation has suggested exposure to minor Group A HRV is significantly associated with exacerbations (Denlinger et al., 2011), and a recent study by Schuler et al. (2014) suggests the differential receptor usage between major and minor Group A HRV may be a mechanism for the observed variation in response to different HRV serotypes, though additional studies of multiple HRV serotypes are required.

## Conclusion

This study demonstrates there are clear differences in the timing of viral protein production, host protein cleavage and host protein mislocalization, as well as in the amount of viral RNA produced, during HRV2 and HRV16 infection. The mislocalization of some nuclear proteins (SC35 and Sam68) occurs early after the initial infection, prior to the complete disruption of the nuclear pore. Other nuclear proteins (hnRNP-C1/C2) seem to require complete disruption of the nuclear pore, which may be a consequence of this protein carrying a strong nuclear retention signal. Future work is aimed at further investigating the differences between different HRV serotypes and determining whether the variation observed between HRV2 and HRV16 is similar for other major and minor Group A HRV serotypes.

## Conflict of Interest Statement

The authors declare that the research was conducted in the absence of any commercial or financial relationships that could be construed as a potential conflict of interest.

## Supplementary Material

Figure S1 **Human rhinovirus effects in A549 cells. (A,B)** A549 cells were infected without (mock) or with HRV2 or HRV16 (MOI of 5) for 24 h and cells lysed collected and subjected to Western analysis as described in **Figure 1**. The approximate protein size (kDa) is shown on the left and the specificity of the antibodies is indicated on the right. Where cleavage products are observed, bands corresponding to full length proteins are indicated with arrowheads and cleavage products are indicated with brackets. cp – cleavage products. **(C)** A549 cells were grown on coverslips and infected without (mock) or with HRV2 or HRV16 (MOI of 5). At 24 h p.i. cells were fixed, permeabilized and probed with anti-dsRNA antibodies, followed by CF488 secondary antibodies. Coverslips were mounted in ProlongGold mounting media with DAPI. Fluorescence was imaged by CLSM (see Materials and Methods).

Figure S2 **HRV2 and HRV16 replicate in A549 cells**. Real-time PCR was used to assess the quantity of viral RNA present in infected cells at 24 h p.i. for HRV2 and HRV16 infection. Data is shown as arbitrary units relative to GAPDH.

Figure S3 **Cell death after HRV2 or HRV16 infection**. Ohio-HeLa cells were grown in six well plates and were mock infected, or infected with HRV2 or HRV16, at an MOI of 1. Live cells were imaged at 24 h p.i. using a Leica DMIL light microscope.
