# Supplementary figures and images for: Variation in the nuclear effects of infection by different human rhinovirus serotypes
Source: Front Microbiol. 2015 Aug 24;6:875. doi: 10.3389/fmicb.2015.00875 (PMC4547043; doi:10.3389/fmicb.2015.00875)

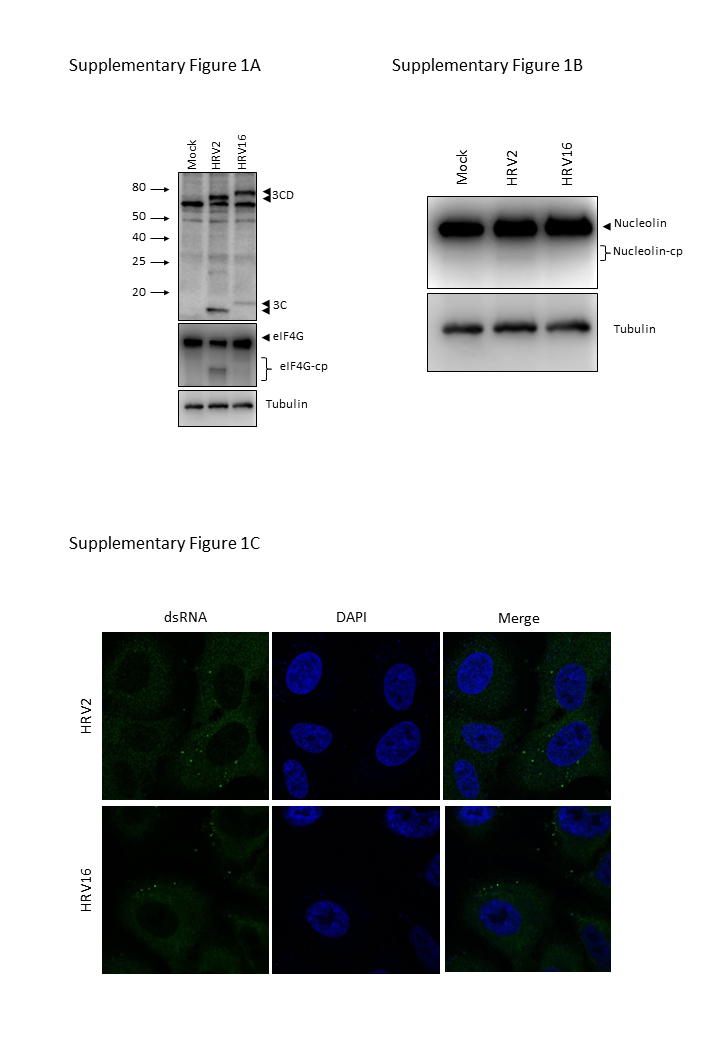

Supplement: Figure S1 — Human rhinovirus effects in A549 cells. (A,B) A549 cells were infected without (mock) or with HRV2 or HRV16 (MOI of 5) for 24 h and cells lysed collected and subjected to Western analysis as described in Figure 1. The approximate protein size (kDa) is shown on the left and the specificity of the antibodies is indicated on the right. Where cleavage products are observed, bands corresponding to full length proteins are indicated with arrowheads and cleavage products are indicated with brackets. cp – cleavage products. (C) A549 cells were grown on coverslips and infected without (mock) or with HRV2 or HRV16 (MOI of 5). At 24 h p.i. cells were fixed, permeabilized and probed with anti-dsRNA antibodies, followed by CF488 secondary antibodies. Coverslips were mounted in ProlongGold mounting media with DAPI. Fluorescence was imaged by CLSM (see Materials and Methods). [file Image_1.TIF]

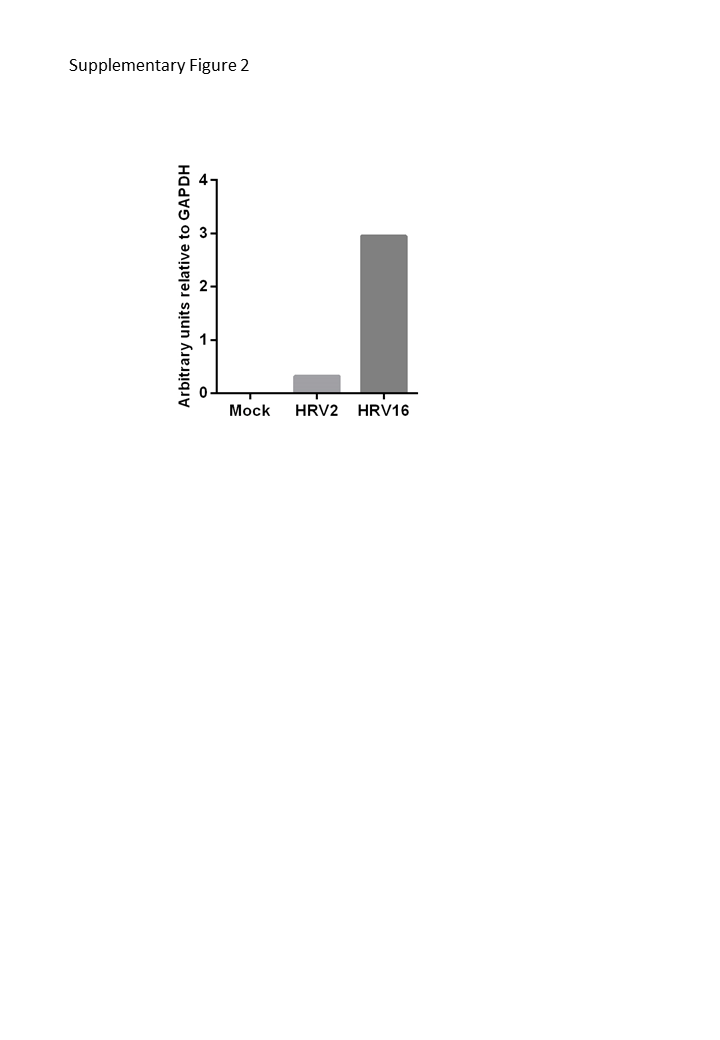

Supplement: Figure S2 — HRV2 and HRV16 replicate in A549 cells. Real-time PCR was used to assess the quantity of viral RNA present in infected cells at 24 h p.i. for HRV2 and HRV16 infection. Data is shown as arbitrary units relative to GAPDH. [file Image_2.TIF]

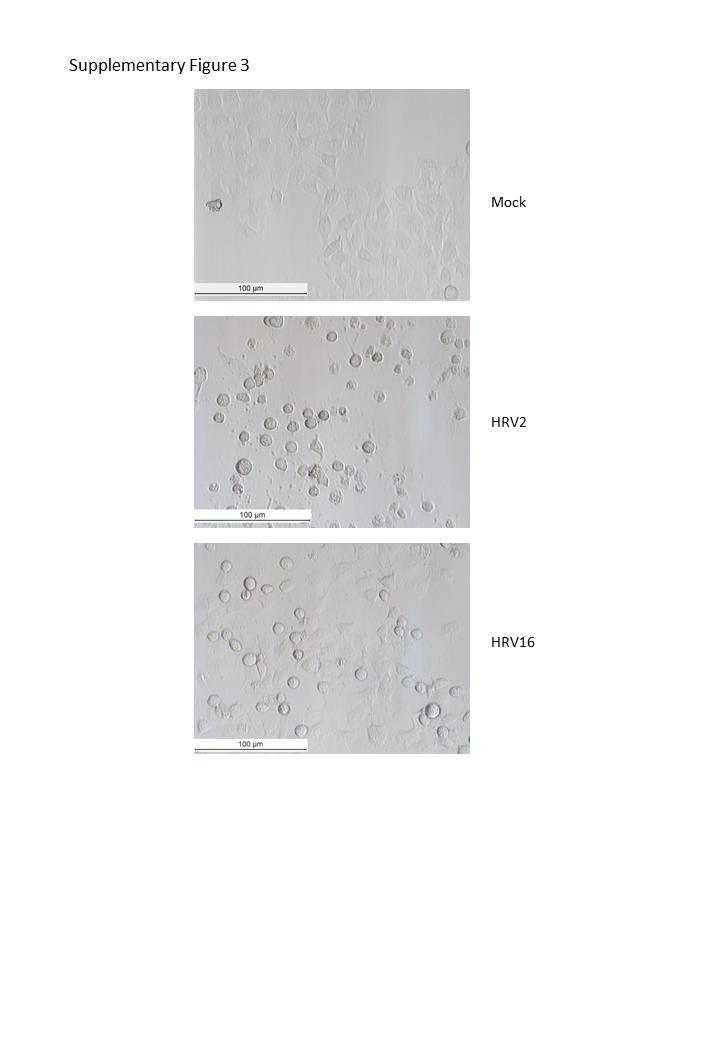

Supplement: Figure S3 — Cell death after HRV2 or HRV16 infection. Ohio-HeLa cells were grown in six well plates and were mock infected, or infected with HRV2 or HRV16, at an MOI of 1. Live cells were imaged at 24 h p.i. using a Leica DMIL light microscope. [file Image_3.TIF]
